# Effectiveness of Daily Mindfulness Meditation App Usage to Reduce Anxiety and Improve Well-Being During the COVID-19 Pandemic: A Randomized Controlled Trial

**DOI:** 10.7759/cureus.42432

**Published:** 2023-07-25

**Authors:** Katie O'Donnell, Melanie Dunbar, Diana Speelman

**Affiliations:** 1 Obstetrics and Gynecology, Lake Erie College of Osteopathic Medicine, Erie, USA; 2 Behavioral Health, Lake Erie College of Osteopathic Medicine, Erie, USA; 3 Biochemistry and Medical Genetics, Lake Erie College of Osteopathic Medicine, Erie, USA

**Keywords:** well-being, mindfulness, meditation, covid-19 pandemic, anxiety

## Abstract

Introduction

This study examined the effect of 10-minute daily meditation app usage for 30 days on adult anxiety and mental well-being during the first year of the COVID-19 pandemic.

Methods

Participants were randomized into intervention (10 minutes of daily usage of the Insight Timer app) or control groups. Participants completed surveys to assess anxiety and well-being pre- and post-study. Data were analyzed using t-tests, analysis of variance (ANOVA), or nonparametric equivalents.

Results

Pre-study results were comparable between groups. The pre- vs. post-study General Anxiety Disorder-7 (GAD-7) scores for anxiety decreased in the intervention group (n=18, median 5.5 vs. 3.0 (pre vs. post), p=0.0233, d=0.50), but not in the control group (n=28). The intervention group had a lower median GAD-7 score than the control group post-study (3.0 vs. 8.0, p=0.0223, d=0.35). Pre- vs. post-study mean 5-item World Health Organization Well-Being Index (WHO-5) scores were improved in both the control (11.6 vs. 12.9 (pre vs. post), p=0.0408, d=0.36) and intervention groups (12.0 vs. 16.3 (pre vs. post), p=0.0001, d=0.77), although it was higher in the intervention group (16.3 vs. 12.9, p=0.0056, d=0.88).

Conclusion

Ten minutes of daily meditation app usage for 30 days may reduce anxiety and improve well-being in adults during the COVID-19 pandemic.

## Introduction

The COVID-19 pandemic resulted in disrupted daily routines for people worldwide, with the potential to have a negative impact on mental health. In March 2020, extensive research was underway on the pathophysiology and treatment of this novel coronavirus and COVID-19 [1-3], but fewer studies were focused on methods for dealing with the overwhelming stress and emotions that accompany a pandemic and the disruptions to daily life. This necessitated the exploration of potential nonpharmacologic interventions for coping with anxiety and maintaining or improving well-being during this time.

The concept of “mindfulness” is generally accepted as simply being aware in the present moment [4]. The use of mindfulness and meditation smartphone apps are relatively new developments that offer more accessibility of these techniques to anyone with a smartphone [5]. Thus, it is imperative that studies explore the effectiveness of this resource at our fingertips, particularly with respect to the impact that the use of a mindfulness meditation app may have on anxiety and well-being while living during a pandemic.

Mindfulness is currently at the forefront of the emerging nonpharmacologic modalities for managing anxiety in the field of psychiatry [6]. Past studies have demonstrated the use of mindfulness interventions to decrease caregiver burden and improve psychological well-being [7] and help decrease perceived stress in medical students [8]. Kemper et al. demonstrated that a diverse population of health care professionals are interested in online training in focused attention meditation and that brief training periods can improve relaxation, resilience, stress, affect, and flourishing [9]. Wen et al. demonstrated that it is feasible to run a completely virtual study examining the use of a mindfulness smartphone app in medical house staff via online standardized surveys [10]. A myriad of studies have been completed on the effectiveness of meditation apps in various settings; however, many have ties to the companies that produce the apps, creating a potential for bias and conflict of interest. Even so, several of these studies were able to demonstrate that it is feasible to deliver mindfulness training via meditation smartphone apps for virtual studies [11,12].

Today, buzzwords, such as “mindfulness,” “wellness,” “meditation,” and “lifestyle,” abound in popular media. This suggests a clear interest from society in what an established mindfulness practice can contribute to enhancing and optimizing mental health. Considering these interests from the public and combining them with the need for an intervention to mitigate the negative effects that the pandemic could have on mental health, we utilized a randomized, controlled trial to investigate the impact of 30 days of mindfulness meditation app usage on anxiety, mental well-being, and general health trends in adults 18 years and older during the first year of the COVID-19 pandemic.

## Materials and methods

The study was approved by the Lake Erie College of Osteopathic Medicine Institutional Review Board (LECOM IRB, approval number: 27-126), and the protocols were registered at the ClinicalTrials.gov website (No. NCT04369378). The aim of this study was to determine the impact of daily mindfulness mediation app usage for 30 days via a randomized, controlled trial.

Participant recruitment and consent

Participants were recruited via registration on the ClinicalTrials.gov website, LECOM social media accounts, advertisement on the LECOM website, and emails sent from the principal investigator to students, faculty, and staff associated with LECOM. The first subject was enrolled on August 19, 2020, and the last subject was enrolled on February 24, 2021. The study completion date was May 24, 2021.

Participant inclusion and exclusion

Inclusion criteria stated that participants must be 18 or older to participate in this study. The participants additionally needed access to a smartphone and ability to download the mindfulness app, fluency in English, and the ability to complete surveys independently. Exclusion criteria included current regular use of a mindfulness or meditation app, regular practice of mindfulness or meditation, regular therapy sessions, inability to complete surveys independently, or any mental health restrictions that would prevent them from participating.

Study design, participant attrition, and protocol deviations

This interventional study was designed as a randomized controlled trial. A parallel assignment intervention model for a 1:1 equivalence allocation ratio was used. Allocation to each group was determined using a random sequence generator with a 1:1 allocation ratio in blocks of eight (https://www.sealedenvelope.com/simple-randomiser/v1/lists). The participants were assigned an ID number to fill out the questionnaires throughout the study so that de-identified information could be collected from the study participants. Adults over the age of 18 were randomly assigned to 10 minutes of daily use of the Insight Timer (Insight Network Inc., USA) meditation app (intervention) for 30 days or to the control group (no usage of the meditation app). Two days before the end of this period, the participants were sent a link to a Google Form (Google LLC, Mountain View, California, United States) for the post-intervention survey and were asked to complete it within three days of completing the 30-day period. Instructions were also provided to the participants stating that they could now use the app at their discretion regardless of the original group they were assigned to. Two months after the conclusion of the 30-day intervention period (90 days after the study began), the participants were sent a link to a Google Form for a final post-intervention survey. No masking was used during the set-up (open label for participants).

The primary outcome measures were anxiety, as assessed by General Anxiety Disorder-7 (GAD-7) survey questions, and well-being, as assessed by the 5-item World Health Organization Well-Being Index (WHO-5) survey questions, immediately following the 30-day intervention period. Secondary outcome measures included anxiety and well-being two months after the 30-day intervention period, future outlook and hopefulness, sleep habits, nutrition habits, exercise habits, and anticipated and actual continued usage of the meditation app.

**Figure 1:**
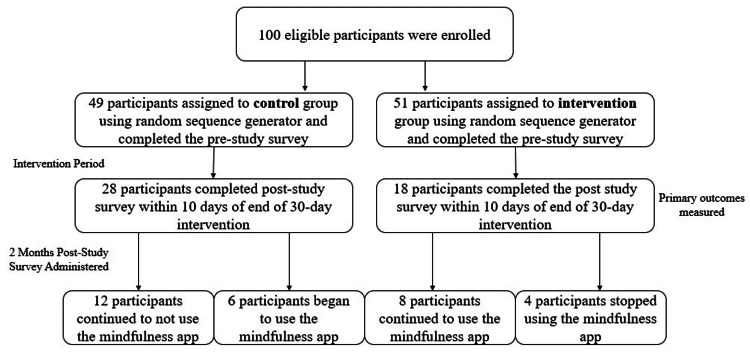
Participant recruitment, group assignment, and attrition over the course of the study period The participants were recruited via email advertisements, social media posts, school website advertisement, and registration on the ClinicalTrials.gov website. The participants were randomly assigned to either the control group or intervention group. Intervention consisted of using the Insight Timer meditation app 10 minutes a day for 30 days. The primary outcomes of the General Anxiety Disorder-7 (GAD-7) and 5-item World Health Organization Well-Being Index (WHO-5) scores were recorded and analyzed at the time of administration of the pre-study survey and the post-intervention surveys. Surveys also included questions about future outlook and hopefulness, sleep habits, and nutrition and exercise habits.

Of the 147 respondents assessed for eligibility, 47 were excluded for declining to participate (did not complete the initial pre-study survey). Of the 100 eligible respondents, 49 were allocated to the control group and 51 to the intervention group using the random sequence generator (Figure 1). Attrition during the 30-day intervention period included failure to complete the daily 10-minute meditation app usage and loss of follow-up (failure to complete the post-study survey or to complete it within a seven-day window at the end of the 30-day intervention period). Attrition during the two-month post-intervention period was due to the lack of follow-up (failure to complete the second post-study survey within a seven-day window).

Protocol deviations were as follows: In the control group, three participants completed the post-intervention survey within 10 days and were included in the data analysis. In the intervention group, one participant completed the post-intervention survey within 10 days and was included in data analysis. All other participants in both groups completed the survey within seven days of the conclusion of the intervention period, with the majority (86%) completing it within the requested three-day window.

Mindfulness meditation app

Insight Timer is a free app that can be downloaded from any app store on a smartphone; it was chosen for this study due to the quantity of meditations it provides without needing a subscription. The app offered guided audio meditations that involved techniques, such as guided breathing, guided imagery, body scanning, gratitude, and affirmations. The participants in the intervention group were given instructions on how to access the Insight Timer app and were instructed to use it for 10 minutes daily for 30 days. The participants were able to choose any meditation. Ten minutes was selected for feasibility in daily usage and to increase the likelihood of compliance; this length of daily meditation for 30 days has previously been shown to be sufficient to increase mindfulness, positive affect, and improve psychosocial outcomes using a similar app [10-12]. Participant usage was self-reported. 

Data collection

Participant ID number and associated email list were stored in a secure physical location within the Principal Investigator’s office (Dr. Diana Speelman), separate from the data. De-identified data were collected via Google Forms, using participant ID numbers for data analysis by group. The investigator completing data analysis (Katie O'Donnell) was blinded to the participant intervention group until after data analysis was completed.

Assessment of anxiety and well-being

Primary outcomes measured were levels of anxiety and well-being before and after the 30-day intervention period. Anxiety levels were assessed by survey questions from the GAD-7 questionnaire when the participants completed the pre-study, post-intervention, and two-month post-study surveys. This metric was chosen based on its validity and standardization [13]. Well-being was assessed by survey questions from the WHO-5 questionnaire at the same time points as mentioned above. This parameter was chosen based on known validity, and it has been used in similar studies to demonstrate self-reported subject mental well-being [14,15]. All the questions from the GAD-7 and WHO-5 questionnaires were incorporated into the pre-, post-intervention, and final two-month post-study surveys and were extracted after the completion of the study for analysis.

Assessment of nutrition, sleep, and exercise habits

As an exploratory investigation secondary to the primary outcomes, investigators created questions for the surveys to collect preliminary data about general lifestyle and psychosocial trends of participants’ nutritional habits, amount of sleep, and exercise. Questions in the survey were also included to determine whether participants used this study period to develop a daily practice of mindfulness that they intended to continue after the study ended. These questions were included for the purpose of identifying potential future avenues of research.

Assessment of future outlook and hopefulness

This study examined trends in participant feelings toward the current and future situation of living through a pandemic, as an exploratory investigation to better understand the environmental context of the sample group in the investigation and to collect preliminary data to identify potential future areas of investigation. Topics included but were not limited to opinions on social distancing, how self-motivated they perceive themselves, perceptions of confidence, connectedness to others and society at large, level of loneliness, and hopefulness.

Statistical analysis

The ClinCalc.com sample size calculator (ClinCalc LLC, USA) was used to determine that a minimum sample size of 16 participants per group would be needed to detect a significant difference for a two-point change in the GAD-7 and WHO-5 scores (primary outcome) with a power of 80% and an alpha of .05 (https://clincalc.com/stats/samplesize.aspx). All data were analyzed using the GraphPad Prism 9 software (GraphPad Software, USA). Prior to the statistical analysis, data were checked for normality using the D’Agostino and Pearson test for normality. Datasets were then analyzed using the appropriate parametric (paired or unpaired t test) or nonparametric test (Mann-Whitney or Wilcoxon test). Significance was set at p < .05. To determine the effect size, estimated Cohen’s d was used. The GAD-7 and WHO-5 results from the pre-, post-intervention, and two-month post-study (final) surveys were analyzed by a repeated measures analysis of variance (ANOVA) or Friedman test (nonparametric equivalent). For responses to survey questions that were created by investigators, the mode for each question was determined and reported given the descriptive nature of these questions regarding lifestyle and psychosocial trends.

## Results

Participant demographics

The participants were primarily located in Erie, Pennsylvania, throughout the duration of this study. Table 1 shows the participant demographic information by group, including age ranges, employment status, occupation, and living situation at the initiation of the study.

**Table 1:** Participant demographic data at study initiation.

Participant demographic information		
Age range	Control	Intervention
18-24	11	5
25-34	13	8
35-44	2	1
45-54	0	4
55-64	1	0
65-74	1	0
Employment status		
Employed - on site	9	4
Employed - from home	0	1
Employed - furloughed	0	0
Unemployed	1	0
Student - full time	18	13
Student - part time	0	0
Homemaker	0	0
Military	0	0
Retired	0	0
Unable to work	0	0
Occupation		
Student	15	12
Higher education/teaching	3	2
Healthcare - patient care	4	1
Higher education/support	2	1
Others/prefer not to answer	4	2
Living situation		
With family	12	8
By myself	6	2
By myself with pets	4	1
With roommate(s)	6	7

Decreased anxiety score after daily meditation app usage for 30 days

The participant surveys included questions from the GAD-7 to assess general anxiety. The GAD-7 pre-study scores were not significantly different between the control and intervention groups. The pre- vs. post-study GAD-7 scores did not significantly differ in the control group, but the GAD-7 scores did decrease post-intervention in the intervention group (Table 2; median score 7.7 vs. 3.0 (pre vs. post), p=0.0233, d=0.50). In addition, the intervention group had a lower score than the control group post-study (median 3.0 vs. 8.0 (intervention vs. control), p=0.0223, d=0.35).

**Table 2:** GAD-7 scores and comparisons before and after the 30-day intervention period. *p < .05; GAD-7: General Anxiety Disorder-7

GAD-7 score	Control group pre-study	Intervention group pre-study	Control group post-study	Intervention group post-study	p-value
N	28	18	28	18	
Mean score	8.6	7.7			0.5910
Mean score	8.6		8.0		0.3420
Median score		7.7		3.0	0.0233*
Median score			8.0	3.0	0.0223*

Improved well-being after daily meditation app usage for 30 days

The WHO-5 pre-study scores were comparable (Table 3). The pre- vs. post-study WHO-5 scores were improved in the control group (mean 11.6 vs. 12.9 (pre vs. post), p=0.0408, d=0.36)) and in the intervention group (mean 12.0 vs. 16.3 (pre vs. post), p<0.0001, d=0.77). When comparing the WHO-5 means for the control group and intervention group post-study, the intervention group was significantly better (16.3 vs. 12.9 (intervention vs. control), p=0.0056, d=0.88).

**Table 3:** WHO-5 scores and comparisons before and after 30-day intervention period. * p < .05, ** p < .01, *** p < .005; WHO-5: 5-item World Health Organization Well-Being Index

WHO-5 score	Control group pre-study	Intervention group pre-study	Control group post-study	Intervention group post-study	P-value
N	28	18	28	18	
Mean score	11.6	12.0			0.7499
Mean score	11.6		12.9		0.0408*
Median score		12.0		16.3	0.0001***
Median score			12.9	16.3	0.0056**

Anxiety score at the two-month post-study

Due to the post-study attrition limiting the statistical analysis of the secondary outcomes, only the median scores of each group at the two-month (final) post-study time point are reported here. For those in the control group who continued to not use the meditation app, the GAD-7 median score was 5.42 (n=12); the median score for these participants at the time of the pre-study survey was 8.83 and 6.92 at the time of the post-intervention survey (Table 4). For those in the control group who then chose to use the app after the 30-day no-intervention period, the GAD-7 median score was 7 (n=6); at the time of the pre-study survey, the median score for these participants was 9.17 and 9.0 at the time of the post-intervention survey. For those in the intervention group who continued to use the meditation app after the 30-day intervention, the GAD-7 median score was 3.5 (n=8); these participants had a median score of 6.5 at the time of the pre-study survey and 3.25 at the time of the post-intervention survey. For those in the intervention group who stopped using the app after the 30-day period, the median GAD-7 score was 5.75 (n=4); at the time of the pre-study survey, the median score for these participants was 8 and 8.25 at the time of the post-intervention survey. 

**Table 4:** Mean GAD-7 and WHO-5 scores broken down after the two-month post-study survey. GAD-7: General Anxiety Disorder-7; WHO-5: 5-item World Health Organization Well-Being Index

Group (n)	GAD-7 mean score pre-study	GAD-7 mean score post-study	GAD-7 mean score 2 months post-study	WHO-5 mean score pre-study	WHO-5 mean score post-study	WHO-5 mean score 2 months post-study
Control group, continued no app use (12)	8.83	6.92	5.42	11.75	13.67	14.5
Control group, app use began after intervention (6)	9.17	9	7	12.83	13.33	13.33
Intervention group, continued app use after intervention (8)	6.5	3.25	3.5	13.63	18.38	17.75
Intervention group, stopped using app after intervention (4)	8	8.25	5.75	14.5	14.5	15.25

Well-being at the two-month post-study

At the two-month post-study time point, those in the control group who continued to not use the app had a mean WHO-5 score of 14.5 (n=12); at the pre-study survey time point, the mean score was 11.75; and at the post-intervention survey time point, the mean score was 13.67 (Table 4). Those in the control group who began to use the meditation app after the 30-day intervention reported a mean score of 13.33 (n=6), whereas 12.83 was the mean score at the pre-study survey time point, and 13.33 was the mean score at the post-intervention time point. Those in the intervention group who continued to use the meditation app after the intervention averaged a score of 17.75 (n=8), while the mean score when completing the pre-study survey was 13.63, and the mean score at the post-intervention survey time point was 18.38. Those in the intervention group who stopped using the app after the 30-day period averaged a score of 15.25 (n=4), and 14.5 was the mean score at both the pre-study and post-intervention survey time points for these participants.

Sleep, nutrition, and exercise habits

Responses to the questions regarding nutritional, sleep, and exercise habits were analyzed as modes by question. These data points were of interest to investigate common lifestyle patterns and attitudes throughout the first year of the COVID-19 pandemic. Data are presented in Table 5, with the mode indicated in bold for each question.

**Table 5:** Health habits. The participants were asked to report how much they agreed with the above statements. Mode of responses is reported in bold.

Health habits												
“How many hours of sleep do you get per night?”												
Hours	4 h	5 h			6 h			7 h			8 h	
Control (pre; post)	0; 1	5; 2			5; 9			16; 13			2; 3	
Intervention (pre; post)	1; 0	1; 1			7; 6			7; 7			2; 4	
“Over the last 2 weeks, how often have you eaten take-out food?”												
Response	1 meal/day or more	1 or 2 meals per week			3 meals per week, not daily			Less than 1 meal per week			Never- only eat homemade	
Control (pre; post)	1; 1	9; 11			6; 5			11; 8			1; 3	
Intervention (pre; post)	1; 0	8; 6			1; 2			6; 8			2; 1	
“When eating, I notice the texture of my food and how it tastes.”												
Likert Scale	Strongly agree		Agree			Neutral			Disagree			Strongly disagree
Control (pre; post)	6; 6		18; 15			2; 5			1; 2			1; 0
Intervention (pre; post)	5; 5		5; 8			3; 3			5; 2			0; 0
“In a typical week (or in the last 2 weeks), how often do your meals include fresh fruits, fresh vegetables, and whole grains?”												
Response	1 meal/day or more			3 meals per week or more, but not daily			1 or 2 meals per week			Less than 1 meal per week		
Control (pre; post)	13; 12			15; 14			0; 2			0; 0		
Intervention (pre; post)	10; 11			6; 6			2; 1			0; 0		
“In a typical week, I exercise (walking around your neighborhood counts!)”												
Minutes	> 150			90-150			> 30, < 90			< 30		
Control (pre; post)	5; 4			9; 8			7; 8			7; 8		
Intervention (pre; post)	10; 10			1; 3			5; 3			2; 2		

Future outlook and hopefulness

The participants in both the control and intervention groups at the pre- and post- survey time points most commonly reported that they “agree” with the following statements: “I can motivate myself to make changes in my life if necessary,” “I am confident in myself and my abilities,” “I feel connected to others,” “I feel a sense of control over my life and actions, even if I can’t control what is happening around me,” “Although I do not know what the future holds, I am comfortable with where I am in the present,” “I am able to find the good in every situation,” and “I know that I am one of many living through a global pandemic and recognize that I am still connected to my community even though I might not see them every day” (Table 6). With the participants in the intervention group, more were likely to indicate that they agreed or strongly agreed with the statement “Over the last 2 weeks, I have felt fully present in daily life and live ‘in the moment’” after the intervention, compared with prior to the 30-day period when most responded that they were neutral about this statement. Similarly, most in the control group responded that they disagreed with this statement before the study but agreed with it after the 30-day period. Following the intervention period, more participants in the intervention group responded that they agreed with the statement “I acknowledge experiences without having to judge them as good or bad” compared with prior to the study. The participants in the control group were more likely to disagree with this statement at the end of the 30-day period.

**Table 6:** Future outlook and hopefulness. The participants were asked to report how much they agreed with the above statements. Mode of responses is reported in bold.

Future outlook and hopefulness pre-study and post-study					
“I can motivate myself to make changes in my life if necessary”					
Likert scale	Strongly agree	Agree	Neutral	Disagree	Strongly disagree
Control (pre; post)	6; 6	15; 15	6; 5	1; 2	0; 0
Intervention (pre; post)	4; 7	12; 11	2; 0	0; 0	0; 0
“I am confident in myself and my abilities”					
Control (pre; post)	4; 5	14; 15	6; 3	3; 5	1; 0
Intervention (pre; post)	1; 5	11; 10	2; 3	4; 0	0; 0
“I feel connected to others”					
Control (pre; post)	5; 4	11; 13	9; 10	3; 1	0; 0
Intervention (pre; post)	4; 5	9; 10	5; 2	0; 1	0; 0
“I feel a sense of control over my life and actions, even if I can't control what is happening around me.”					
Control (pre; post)	1; 4	10; 11	5; 9	5; 4	0; 0
Intervention (pre; post)	4; 6	9; 10	3; 0	2; 1	0; 1
“Although I do not know what the future holds, I am comfortable with where I am in the present.”					
Control (pre; post)	9; 5	8; 15	6; 4	5; 3	0; 1
Intervention (pre; post)	4; 6	5; 8	5; 3	4; 1	0; 0
“Over the last 2 weeks, I have felt fully present in daily life and live “in the moment”					
Control (pre; post)	1; 1	10; 13	6; 8	11; 6	0; 0
Intervention (pre; post)	2; 7	3; 7	10; 3	3; 1	0; 0
“When having a conversation with others, I am able to fully listen to what is being said instead of planning what I am going to say next”					
Control (pre; post)	3; 5	11; 11	8; 8	5; 3	1; 1
Intervention (pre; post)	0; 3	10; 10	5; 3	3; 2	0; 0
“I acknowledge experiences without having to judge them as good or bad”					
Control (pre; post)	4; 4	10; 8	7; 6	6; 9	1; 1
Intervention (pre; post)	0; 3	6; 9	6; 3	5; 3	1; 0
“I am able to find the good in every situation”					
Control (pre; post)	3; 4	16; 15	5; 5	4; 4	0; 0
Intervention (pre; post)	2; 6	7; 9	5; 3	4; 0	0; 0
“I know that I am one of many living through a global pandemic, and recognize that I am still connected to my community even though I might not see them every day”					
Control (pre; post)	5; 4	12; 13	7; 7	3; 3	1; 1
Intervention (pre; post)	3; 7	12; 9	3; 2	0; 0	0; 0
“I have felt feelings of loneliness frequently over the last 2 weeks”					
Control (pre; post)	3; 5	9; 10	5; 3	8; 8	3; 2
Intervention (pre; post)	3; 0	3; 2	3; 2	6; 6	3; 8
“I am hopeful for the future”					
Control (pre; post)	8; 10	17; 14	2; 2	1; 1	0; 1
Intervention (pre; post)	9; 10	7; 4	1; 3	1; 0	0; 1

Feasibility of continuing a daily mindfulness practice

Before beginning the intervention period, the participants were asked why they were interested in mindfulness and were able to choose multiple responses. The most popular answers in the control group were to “reduce stress,” “reduce anxiety,” and “improve performance.” The most common responses from the intervention group were “reduce stress,” “reduce anxiety,” and “improve performance” and “better sleep.”

After the completion of the 30-day study period, two-thirds of the participants who were originally in the intervention group continued to use the app and completed the final survey. The frequency of app usage varied, with “once daily,” “three times a week,” and “once a month” as the most popular responses for those who continued to use the app after the intervention (Table 7). The mode for length of time of the mindfulness sessions for these participants was “10 minutes.”

One-third of the participants who were originally in the control group began using the app and completed the final survey. The mode for frequency of app usage in the control group who began using the app after the intervention was “once a week.” The mode for length of time that mindfulness sessions lasted per sitting was “5 minutes” for these participants.

**Table 7:** Feasibility of mindfulness app practice. The participants were asked to report how much they agreed with the above statements. Mode of responses is reported in bold.

Feasibility of mindfulness app practice															
“How often do you find yourself using the smartphone app?”															
Frequency				Once daily		3 times a week		Once a week		Once every 2 weeks		Once a month			Never
Control group, began app use				1		1		4		0		1			0
Intervention group, continued app use				2		2		1		1		2			0
“If you still use the smartphone app, how long are your mindfulness sessions per sitting?”															
Frequency	Less than 1 minute	1 minute	3 minutes		5 minutes		10 minutes		15 minutes		20 minutes		25 minutes or more	Not applicable- I stopped using the app	
Control group, began app use	0	0	2		4		0		0		0		1	0	
Intervention group, continued app use	0	0	0		1		5		2		0		0	0	

## Discussion

The negative impact of the COVID-19 pandemic on mental health necessitated interventions to mitigate the psychological impact on persons worldwide and in a manner that is widely accessible. In this study, we investigated the effect of 10 minutes of daily meditation app usage for 30 days on measures of anxiety and mental well-being during the first year of the COVID-19 pandemic. We found that daily usage resulted in a significant decrease in anxiety as assessed by the GAD-7 (medium effect size) and an increase in mental well-being as assessed by the WHO-5 (medium-to-large effect size). No significant improvement in anxiety was observed in the control group. Although both groups had a significant improvement in mental well-being, there was a greater increase in the WHO-5 score in the intervention group compared to the control group (4.3-point mean increase compared with the 1.3-point mean increase; d=0.77). Together, these data support the effectiveness of improving mental well-being and decreasing anxiety with just 10 minutes of daily meditation app usage for 30 days during the COVID-19 pandemic. 

Impact of the COVID-19 pandemic, social distancing, and isolation on mental health

The COVID-19 pandemic, and in particular the social distancing and isolation precautions taken to limit the spread of the novel coronavirus during the first year of the pandemic, created a novel set of stressors that many individuals had not before experienced. The amount of diagnosable depression and anxiety disorders increased in prevalence in 2020 during the pandemic [16], with confinement or isolation in particular having a negative impact on psychological health [17]. These effects were not mediated by population density, as similar psychological impact was observed in rural and urban settings alike [18]. With this pervasive increase in stress associated with the pandemic and the negative impact on mental health and well-being, it was necessary to identify effective, accessible modalities for managing or reducing anxiety and for maintaining or improving mental well-being. Mindfulness or mindful meditation has the potential to serve such a modality for this purpose [19-21], and it is accessible to many in the format of a smartphone app [10,12,22].

Impact of mindfulness on anxiety and mental health during the COVID-19 pandemic

In a study on adults in Italy, lockdown isolation correlated with decreased mindfulness, and with that, increased distress and sleep problems [23]. However, people who score high on trait mindfulness or possess mindfulness skills have reduced stress and anxiety, suggesting that interventions to improve mindfulness have the potential to reduce anxiety related to COVID-19 and improve mental well-being [24-30]. Current practitioners of mindfulness have reduced distress and GAD-7 scores [31, 32], and two weeks of increased mindfulness training was found to reduce depression and anxiety, as was the increased frequency of practice [31]. An eight-week course in mindful meditation with female teachers in Italy during the COVID-19 pandemic resulted in improvement in the measures of anxiety, depression, affective empathy, and psychological well-being, especially in low-resilience individuals [20]. In a larger study population of 476 teachers who adopted online teaching during the pandemic, mindfulness was found to be a significant moderator to decrease emotional exhaustion associated with this shift in teaching modality and improve online teaching efficacy [33]. These findings and similar findings in other studies underscore the use of mindfulness as a self-help tool to help manage and reduce anxiety and improve mental well-being [21,34-36].

Our results demonstrated that 10 minutes of daily meditation app use for 30 days during the early days of the COVID-19 pandemic significantly improved WHO-5 scores, indicating improved mental well-being. One surprising finding of this study was that control group WHO-5 scores also significantly improved post-study compared to pre-study. This could be due to the lessening of lockdown restrictions occurring simultaneously at that time in the pandemic. However, there was a greater increase in the WHO-5 scores in the intervention group compared to the control group after the intervention period, demonstrating that the participants who developed a meditation practice had significantly higher well-being scores than those who did not use the app. Since recent studies have identified a negative impact of the pandemic on mental well-being, as assessed by the WHO-5 during the pandemic and compared with pre-pandemic [37-40], it is important to identify effective tools for improving mental well-being during the present and any future pandemics. This is particularly important since the impact may last beyond the end of lockdown measures [41].

Our results also indicated significantly reduced GAD-7 scores in the participants who used the app for 10 minutes daily for 30 days. These findings support the use of a mindfulness meditation app to cope with mental stressors during a pandemic. The GAD-7 has been used in several studies examining anxiety levels throughout the COVID-19 pandemic [40-42]. In another study involving 10 minutes of mindfulness practice but over a period of 10 days, an increase in positive affect in the study population was found, but no change in negative affect, anxiety, or depression was noted [43]. This difference in effect on anxiety may be related to the shorter duration of the intervention, difference in study population, difference in app and mindfulness guidance used, or other factors. In another study, a four-week randomized controlled trial involving a mindfulness intervention in social workers found no significant change in stress or anxiety, but improved psychological flexibility and self-compassion while decreasing depression in this population [44]. While no effect was found on anxiety in that study, possibly related to the study population and nature of the participants’ work, it is worth noting that the mindfulness intervention still had a positive impact even if in different ways.

Varying aspects of mindfulness may influence the outcomes, as may the interaction between specific populations, stressors, and aspects of mindfulness. Decentering, in particular, was uniquely associated with decreased worry and stress and improved measures of mental health and quality of life in an adolescent population [45]. In a study with health professions students, focus, appreciation, cognitive decentering, and nonreactivity mindfulness skills were found to help participants cope with stress during the COVID-19 pandemic [19]. The acceptance component of mindfulness was also found to play a key role in influencing sleep quality in adults [23]. In a study of college students during the pandemic, an online, group-based mindfulness intervention was found to improve three out of six aspects of psychological well-being, namely, self-acceptance, autonomy, and environmental mastery [46]. Interestingly, a survey-based study in Switzerland found that while mindfulness improved initial resilience, physical activity helped to maintain resilience over time [47]. The latter findings underscore the integration of mental and physical health and the ability of physical health to support mental health.

Having trait mindfulness or developing a mindfulness practice may also be protective against the adverse effects of COVID-19 news exposure [43], social media exposure on psychological distress mediated by rumination in college students [48], and protective against increased tobacco and alcohol use in hospital workers during the COVID-19 pandemic [49]. Together, these studies highlight the negative impact of social media and news exposure on mental health during the COVID-19 pandemic and highlight the potential benefit of mindful meditation practice to attenuate these effects and reduce the likelihood of turning to less healthy coping mechanisms.

Feasibility of using a virtual medium for delivery of mindful meditation guidance

The results of a recent anonymous survey indicated that 68.8% of respondents had participated in mind-body activities since the start of the pandemic, underscoring the public’s interest in nonpharmacologic modalities to manage stress [50]. The use of a virtual medium to deliver guidance for mindfulness or mindful meditation is convenient and widely accessible to anyone with a smartphone [10,12,22]. Even a single session or brief intervention may provide some benefit in the reduction of stress and anxiety [51-53]. By using a free smartphone app, the practice of mindfulness or mindful meditation becomes accessible to anyone with a smartphone, day or night, even in the presence of isolation and lockdown measures as experienced during the COVID-19 pandemic.

Following the intervention period in our study, when the participants were able to choose whether to begin use of the app (if in the control group) or continue use of the app (if in the intervention group), several trends were noted. Those who continued to use the app maintained a lower GAD-7 score and improved WHO-5 score, as compared with the pre-study values. This suggests that these eight participants found the daily practice to be beneficial and so continued to use the app. For the participants who stopped using the app, their pre- and post- study scores did not show improvement, but their final scores did both indicate improvement, suggesting that this particular intervention may not have been beneficial to these four individuals and factored into their decision to stop using the app. The participants in the control group who decided not to use the app following the study saw improvements in both their GAD-7 and WHO-5 scores, possibly reflecting that the 12 individuals achieved improvements in these measures of mental health by other means and did not feel that they would further benefit from use of the app. Of the six participants that decided to begin using the app, there was a slight improvement in GAD-7 scores. Notably, the participants who began using the app after the intervention period most often reported use of the app for five minutes once per week, as compared with the participants who continued using the app and reported use of the app for 10 minutes three times or more each week. The slight decrease in GAD-7 scores in those in the control group who began using the app, therefore, may be reflective of the frequency and duration of meditation app usage. Together, our findings support the feasibility and effectiveness of using a mindfulness app to improve well-being and reduce anxiety in those who are receptive to this type of intervention. 

Diet, exercise, sleep, and future outlook in relationship to using a meditation app

The data from our study regarding nutritional, exercise, and sleep habits lay the groundwork for future research to determine if app-mediated mindfulness practice can influence people to make healthier lifestyle choices. One trend noted was a decrease in the amount of take-out food consumed in the intervention group but an increase in take-out food in the control group after the intervention period. Such acute changes in the diet can be hypothesized to be a result of food shortages and fear of food insecurity, changes in methods of food acquisition, or even changes in motivation to prepare meals during the first year of the pandemic [54]. Although changes in sleep and exercise habits were not noted in our study, such changes were found in other studies [23,36,42,55,56]. For questions related to future outlook and hopefulness, the intervention group demonstrated a trend toward increased acknowledgment of experiences as they are, rather than feeling the need to judge them as good or bad. This suggests that one way in which meditation app-mediated improvements in mental well-being may occur is through acceptance and decentering. These preliminary findings on nutritional habits and future outlook warrant further investigation.

Limitations

The limitations of this study include a relatively small sample size, limited diversity in age and occupational status, high rate of attrition during the intervention period, self-reporting on surveys, and self-motivation regarding use of app and adherence to the study timeline. The changing nature of COVID restrictions and lockdown protocols may have affected participant perception of anxiety and well-being, depending on where the participant was located within the US during the study period. In addition, the first pandemic year when each participant utilized the meditation app may have also affected the perceived anxiety and well-being in the participants. Finally, most participants were affiliated with a single health system; therefore, further studies with other populations are necessary before generalizing the findings.

## Conclusions

Meditation apps can be used at any time by anyone with a smartphone. Daily use of a meditation app during a pandemic may significantly improve well-being and reduce anxiety, with a medium to large effect, which may help to counteract negative impacts on mental health. Further research should be done to explore the link between meditation practice guided by a mobile app and its long-term mental health effects. While meditation app usage may have a positive influence on choices related to diet, sleep, exercise, and future outlook, studies designed to specifically test the influence on each of these are warranted.
